# Assessing Nutrient Removal in Stormwater Runoff for Urban Farming with Iron filings-based Green Environmental Media

**DOI:** 10.1038/s41598-020-66159-7

**Published:** 2020-06-10

**Authors:** Dan Wen, Ni-Bin Chang, Martin P. Wanielista

**Affiliations:** Department of Civil, Environmental, and Construction Engineering, University of Central Florida, Orlando, FL 32816 USA

**Keywords:** Environmental chemistry, Environmental sciences, Pollution remediation

## Abstract

Ensuring urban areas have access to clean drinking water, safe food supply, and uncontaminated water bodies is essential to the good health of millions of urban residents. This paper presents the functionality of Iron Filings-based Green Environmental Media (IFGEM) in terms of nutrient removal efficiencies to support water quality management and urban farming. IFGEM uses recycled materials such as tire crumb and iron filings to help remove nutrients with essential physicochemical properties. In this study, IFGEM were proven effective and sustainable through an isotherm study, a column study of reaction kinetics, and a microstructure examination under various inlet nutrient concentration levels. IFGEMs exhibited over 90% nitrate removal, as well as 50–70% total phosphorus removal, under most inlet conditions. These promising results make IFGEM suitable for treating stormwater runoff, wastewater effluent, and agricultural discharge via varying *ex situ* treatment units in flexible landscape environments. In addition, the byproduct of ammonia generation permits possible reuse of spent IFGEM as soil amendments in crop land, gardens and yards, and green roofs for urban farming. Findings may help secure urban food supply chains and harmonize nutrients, soil, water, and waste management in different urban environments.

## Introduction

Globalization and urbanization have reshaped human society, producing a set of complex, interdependent, and interrelated problems in a food-energy-water-waste nexus under the constraint of limited natural resources^1^. One of the issues facing sustainable development is the contradiction between nutrient (nitrogen and phosphorus) depletion in agricultural production^2^ and nutrient contamination from stormwater runoff, wastewater effluent, and agricultural discharge^3,4^. Undesired nutrient loads to natural systems and the built environment create environmental sustainability concerns in terms of ecosystem degradation due to eutrophication and loss of biodiversity^5,6^. On the other hand, the rapid growth of the global population constantly increases demands for food production, and modern agriculture largely relies on mining-based fertilizers, which are facing a depletion issue, particularly for phosphorus^7^. Through consideration of these intertwined situations, the Environmental Protection Agency^8^ promulgated regulatory standards for remediating the impacts from the non-point source pollution^9,10^ and resolving the contradictory situation in nutrient management.

Best management practices (BMPs) such as filtration basin, grassed swale, green roof, etc., have been used to remediate non-point pollution for decades^11,12^. However, existing BMPs are becoming less effective due to increased nutrient concentrations and runoff volumes, as well as soil contamination via rapid urbanization^13,14^. One solution is to use green sorption media (i.e., media with the inclusion of recycled materials), designed for enhancing nutrient removal both sustainably and cost effectively. However, there are some design complications when attempting to remove multiple nutrients. Phosphorus removal, for instance, mainly depends on high calcium (CaO) content in green sorption media^15,16^, whereas, for nitrogen removal, green sorption media would need clay minerals and tire crumb components following the physiochemical approach^9,10^. The microbiological processes of nitrification and denitrification are also major approaches for nitrogen removal in moisturized environments^17,18^. Thus, the removal processes of phosphorus and nitrogen are relatively separate in green sorption media applications.

Some studies have tried to improve simultaneous nitrogen and phosphorus removal by applying different filtration media layers, one of which is iron-rich sandy loam^19^. One study evaluated biological phosphorus removal through the use woodchips only, as well as through woodchips with activated alumin/gravel mixture. The mixture showed 19 times greater reduction in total phosphorus, while the woodchip was only able to remove reactive phosphorus (bioavailable ones)^20^. Overall, metal-based filtration media (iron, aluminum, calcium, etc.) are more appropriate for phosphorus removal^21,22^ but the nitrogen removal was not considered in this study.

In recent years, nanoscale zero valent iron (NZVI) particles have been studied for their promising removal of nitrate^23,24^ and phosphorus^25,26^. However, NZVI have an intensive reaction rate and may raise some public health concerns when considering their application in retrofitted BMPs^27^. In addition, nano materials are normally expensive for large scale applications. However, iron filings, as an industrial byproduct, have no such concerns. Hence, green sorption media that can simultaneously remove and even recover nitrogen and phosphorus via just physiochemical reactions are more valuable, cost-effective, and sustainable to implement in areas that might not be suitable for microbiological reactions. There might be a potential candidate to be mixed with existing green sorption media known as Bio-sorption Activated Media (BAM) for the enhancement of nitrogen and phosphorus removal from stormwater runoff^9,10,15,18,28–33^.

This study presents a preliminary evalution of the ability of a mixture of iron filings with clay and sand, called Iron-Filings based Green Environmental Media (IFGEM), to provide simultaneous removal of nitrogen and phosphorus via physicochemical processes. To gain a fundamental understanding of how IFGEM mix works and confirms their applicability, the objectives of this study are thus to: (1) conduct an isotherm study for IFGEM to gain understanding of their absorption and adsoption characteristics for simultaneous nitrate and phosphorus removal under neutral pH and room temperature conditions; (2) carry out a column study to test their nutrient removal efficiencies under various influent concentrations; (3) assess the holistic performance of IFGEM with the aid of an imaging analysis technique for discussing their potential for nutrient reuse/recovery.

The research questions to be answered include: (1) how would the iron filings interact with different green sorption media components and what are their impacts on nitrate and phosphorus removal? (2) how would the different initial nutrient conditions affect the reaction kinetics and removal efficiencies through IFGEM? (3) will ammonia be generated in the treatment process due to the reduction effect provided by iron filings, and, if so, how would ammonia affect the performance of IFGEM? And (4) what are the differences between raw and used (e.g., spent) IFGEM in terms of microstructure, which might support the nutrient recovery by using spent IFGEM? We hypothesized that: (1) nitrate reduction would be significant due to the existence of iron filings as an electron donor; (2) ammonia may be produced as a byproduct from nitrate reduction; (3) phosphorus removal would be enhanced due to the precipitation of phosphate when ferrous, ferric ion, and iron oxides are more available; and (4) nutrient removal would be largely impacted by the influent nutrient concentrations.

## Material and Methods

**Figure 1:**
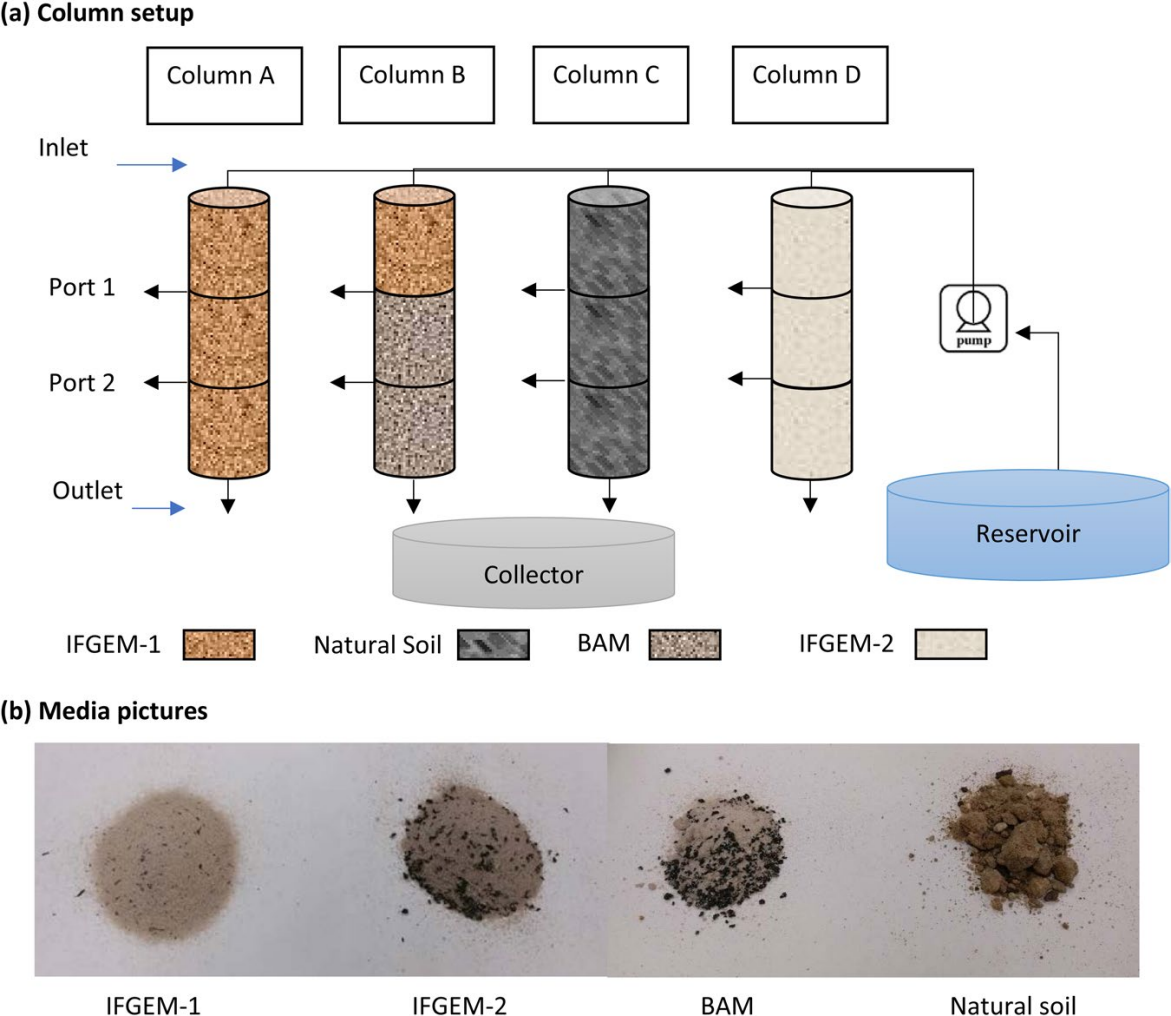
(**a**) diagrams of column setup and (**b**) pictures of media IFGEM-1, IFGEM-2, BAM, and natural soil.

This study was conducted through three different processes (Fig. 1S, Supplementary Materials 1), including an isotherm study, a column study, and material characterization. Two types of IFGEM (denoted as IFGEM-1 and IFGEM-2) were evaluated. The mix of IFGEM-1 is composed of 96.2% sand and 3.8% iron filings by volume, whereas the mix of IFGEM-2 is composed of 80% sand, 10% tire crumb, 5% clay, and 5% iron filings by volume. The differential effect due the inclusion of clay and tire crumb can be realized through this research design. While tire crumb is used simply for regulating the infiltration rate, clay is the key component providing interactions with nitrogen and phosphorus. The isotherm study answer parts of questions 1 and 3, which is critical for determining the absorption capacity and working mechanism. Furthermore, the column study was expected to answer questions 1, 2, and 3. Material characterization would help in answering question 4. Finally, the potential of spent IFGEM for nutrient recovery can be realized. The procedure of statistical analysis for the significant differences between column study scenarios and the details of material characterization are introduced in the Supplemental File.

### Isotherm study

An adsorption isotherm experiment was conducted separately for nitrate and phosphorus in IFGEM-1 and IFGEM-2 with deionized water under neutral pH. Five 500 mL flasks containg 30–120 g media mass were prepared with a 300 mL solution of 1.0 mg/L as total nitrate or phosphorus. The experiment was carried out under room temperature on the rotary shaker at 250 rpm for 1 hour. Then, the water sample from each flask was filtered through 0.45-µm membrane filters before the nutrient analysis. The parameters analyzed were nitrate and ammonia for the nitrate isotherm experiment, and total phosphorus for the phosphorus isotherm experiment. The Freundlich and Langmuir isotherm equations were adopted to analyze the data. The freundlich isotherm was obtained by plotting log q versus log C, and the Langmuir isotherm by plotting 1/q versus 1/C. The following two equations were applied in this study.

Freundlich isotherm equation:

1 $$\log \,{q}_{e}=\,\log \,{K}_{F}+\frac{1}{n}\,\log \,{C}_{e}$$

Langmuir isotherm equation:

2 $$\frac{1}{{q}_{e}}=\left(\frac{1}{{K}_{L}{q}_{m}}\right)\frac{1}{{C}_{e}}+\frac{1}{{q}_{m}}\,$$

where ${C}_{e}$ is the aqueous concentration of adsorbate (mg/L), *q*_*e*_ is the sorbed concentration (mass of absorbed adsorbate/mass adsorbent), *q*_*m*_ is the maximum capacity of adsorbent for adsorbate (maximum mass of absorbed adsorbate/mass adsorbent), *C* is the aqueous concentration of adsorbent (mass/volume), *K*_*L*_ is the Langmuir equilibrium constant, *K*_*F*_ is a constant indicative of the relative adsorption capacity of the adsorbent (mg^1−(1/n)^ L^1/n^ g^−1^), and n is a constant indicative of the intensity of the adsorption.

### Design and setup of column tests

Column experiments were designed to simulate the field treatment conditions with a down-flow strategy, which is critical for addressing a suite of absorption, adsorption, ion exchange, precipitation, and oxidation/reduction reactions between sorption media and nutrients that leads to determining the answers to questions 1, 2, and 3. Four column sets (named from A to D) were constructed with 10 cm (4 inches) diameter PVC pipes, and each column set was divided into three equivalent sections (top, middle, and bottom), and each section had a depth of 30 cm (1 foot) for convenient water sampling. As shown in Fig. 1(a), all three sections of column A were filled with IFGEM-1. In column B, the top section was filled with IFGEM-1, and the middle and bottom sections were filled with BAM. Column C was a control column, and thus was filled with natural soil for all three sections. In column D, all three sections were filled with IFGEM-2. All four columns from A to D were attached to a wooden board where the effluent from the previous section was the influent of the following one and the joints between sections were wrapped with parafilm to eliminate outside impacts. A picture of the four media used is shown in Fig. 1(b).

Distilled water was spiked with nitrate and phosphate standard solutions to three concentration levels (nitrate = 0.6, 1.2, 1.8 mg/L; TP = 0.3, 0.5, 0.7 mg/L) in order to simulate the fluctuation of nutrient concentrations in real stormwater runoff, agricultural discharge, or wastewater effluent from a secondary wastewater treatment plant^34^. The columns were flushed with distilled water for a few days before starting the experiment to wash out any possible dissolvable contaminants, and were also flushed for a few hours each time before operating the columns under a different influent condition for eliminating any possible impacts from the previous influent concentrations. It was expected that physicochemical reduction/absorption would be the main mechanism for nitrate removal instead of microbiological effects, and that the only exception would be column C, which was the control column with natural soil collected from SR35 Basin 2 located in Ocala, Florida. The experiment was conducted at stable room temperature around 23 °C. A pair of peristaltic pumps with a fixed flow rate of 8 mL/min were used to pump the water from a reservoir. The pumping rate is equivalent to the infiltration rate of 1.09 cm/hr (0.43 in/hr), which was set up to mimic the drip irrigation condition using stormwater runoff or agricultural discharge. With this pumping rate, the columns were never fully saturated. The HRT and soil moisture from each section were recorded when the flow rate of the effluents could be stabilized after 3 hours of operation. Water samples were collected in triplicate from the reservoir and outlet of each section. The values of dissolved oxygen (DO), oxidation reduction potential (ORP), and pH were measured immediately after collection. IFGEM media samples were collected before and after the experiment for morphological comparison under a confocal microscope. Nitrate concentrations were analyzed through the HACH kit TN830, ammonia concentrations were analyzed through the HACH kit TN835, and total phosphorus (TP) concentrations were analyzed with the HACH Phosphorus (Total) TNT Reagent Set (summarized in Table 1). All water samples were analyzed within 24 hours after collection.

**Table 1:** Column study sample quality parameters and methods.

Parameter	Method/instrument	Range
pH	Waterproof Double Junction pHTestr^®^ 30	1 to 14
Dissolved oxygen	HACH HQ40D - IntelliCAL LDO101 LDO	0.01–20 mg/L
ORP	HACH HQ40D - MTC101	±1200 mV
Soil moisture	EC-5 SMALL SOIL MOISTURE SENSOR	0–100%
Nitrates	Method 10206	0.05–13.50 mg/L NO3-N
Total phosphorus	DR/800 Method 8190	0.06–3.50 mg/L PO4
Ammonia	Method 10205	0.015–2.00 mg/L NH3-N

#### Kinetic study

Performance in terms of filtration kinetics refers to the efficiency of the treatment process based on the concentration from the effluent and influent . Kinetic study for nitrate reduction and phosphorus adsorption in IFGEM was conducted for the field design and BMP applications. The reaction time is recorded as hydraulic retention time (HRT) from each column section, answering question 2. Equation 3 is a general version of the zero, first, second, or higher order rate equations, which was applied to the kinetic study for determining the best fit reaction orders.

3 $$\frac{dc}{dt}=k{[C]}^{n}$$

where *C* is the concentration of nitrate/phosphorus in solution (mg/L), *n* is the reaction order, and *k* is the reaction constant.

In a zero order reaction, the reaction rate is independent of the concentration of reactants. The reaction rate will not change when the reactants’ concentration is different per se. However, the first-order reaction is a reaction that proceeds at a rate that depends linearly on only one reactant concentration. That is, when the key reactant has a higher concentration, the reaction speed is faster than the case with a lower concentration. Second order reaction proceeds at a rate that depends non-linearly on the power of 2 of the key reactant’s concentration.

## Results

### Material characterization

#### Physical property

**Figure 2:**
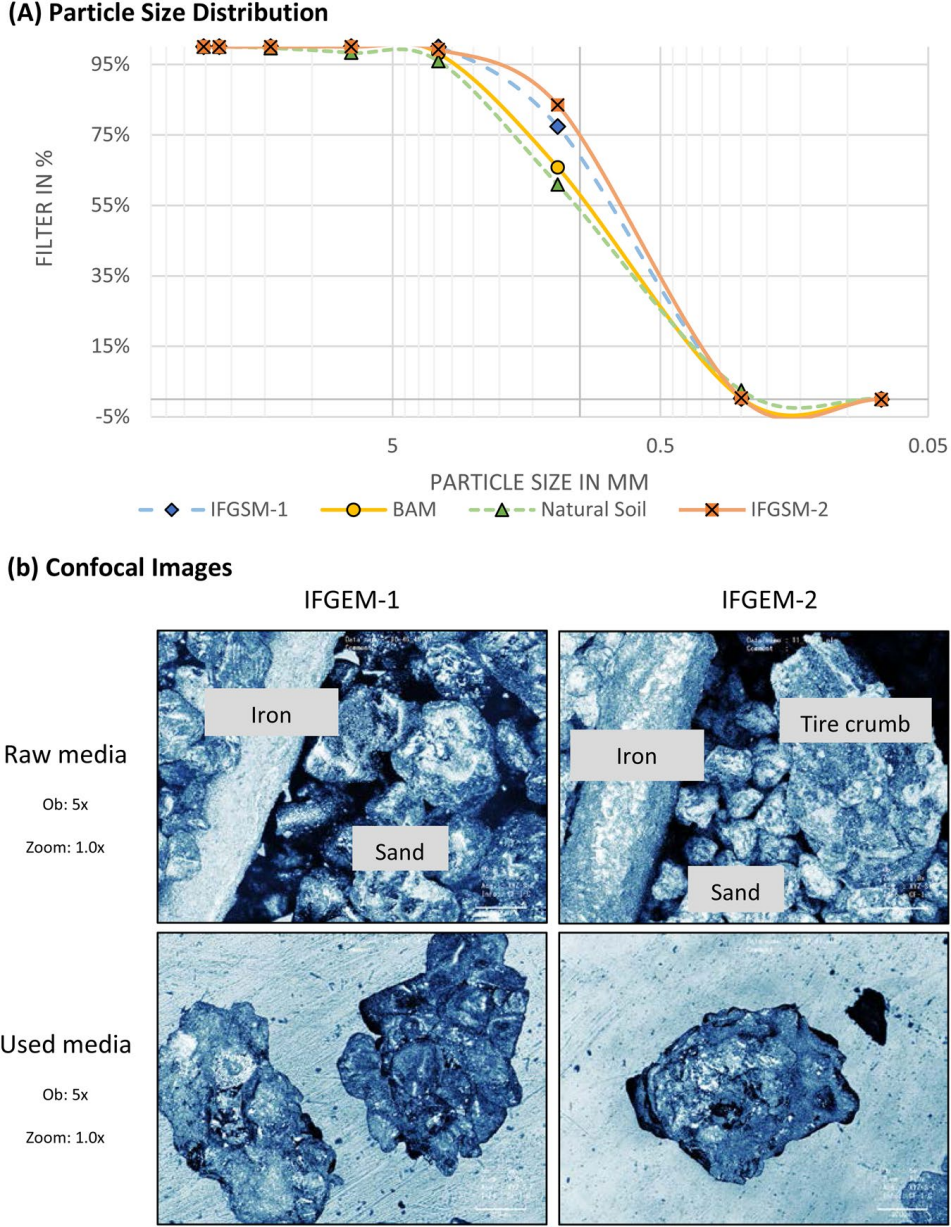
(**a**) Particle size distribution for natural soil and media mixes and (**b**) confocal images raw media for IFGEM-1 shows iron filings and sand and IFGEM-2 shows iron filings, smaller sand, and tire crumb and used IFGEM-1 shows iron filing coated by surrounding materials and used IFGEM-2 shows iron filing coated by surrounding materials.

Figure 2(a) shows the particle size distribution curves of the four types of sorption media. The two IFGEMs showed similar distribution patterns, as opposed to natural soil and BAM. Higher percentages of finer particles were found in IFGEMs due to the existence of fine sand (IFGEM-1) and clay content (IFGEM-2). The physical properties of the four media mixes are shown in Table 2. BAM has the lowest density of 1.39 g/cm^3^ because of the tire crumb, while the densities of IFGEM-1 and IFGEM-2 are 2.73 and 2.60 g/cm^3^, respectively. The soil density of 2.36 g/cm^3^ falls in between the density values of IFGEMs and BAM. Significant differences exist when comparing BET surface areas. Natural soil showed the highest value of 9.3712 m^2^/g because of aggragated clay particles, followed by IFGEM-2, with a value of 1.3963 m^2^/g. BAM and IFGEM-1 exhibited values of 0.7059 and 0.3142 m^2^/g, respectively. This is because IFGEM-1 has no micro particles, such as clay, which usually exhibits a large surface area. Another significant difference among the four media is the infiltration rate. Natural soil showed the lowest value of 0.003 cm/s, while the values of IFGEM-1, BAM, and IFGEM-2 are of the same magnitude as 0.028, 0.026, and 0.017 cm/s, respectively. The porosity differences of the four media were not as significant as the other parameters, ranging from 36.16 to 40.43% (IFGEM-1 <IFGEM-2 <BAM < natural soil).

**Table 2:** Material Characteristics.

	IFGEM-1	IFGEM-2	BAM	Natural Soil
Density (g/cm^3^)	2.73	2.60	1.39	2.36
BET Surface Area (m^2^/g)	0.3142	1.3963	0.7059	9.3712
Porosity (%)	36.16	37.31	40.10	40.43
Hydraulic Conductivity (cm/s)	0.028	0.017	0.026	0.003

#### Morphological changes

The morphological images of pre- and post-treatment IFGEM-1 and IFGEM-2 are shown in Fig. 2(b). The iron filing pieces could be observed clearly in the raw media of both IFGEMs. The tire crumb and smaller sand particle sizes were observed in IFGEM-2 when compared with IFGEM-1. After treatment, the first observable difference was the color, where both IFGEM-1 and IFGEM-2 turned brownish. In addition to the color change, the iron filings could not be observed by the naked eye after treatment, since they were oxidized and coated by surrounding materials. When coated iron was exposed by external forces, it revealed that the size of the iron filings had significantly decreased as it was dissolved and reacted with other particels during the water treatment process.

### Results of isotherm study

#### Phosphorus and nitrate absorption under neutral pH

**Figure 3:**
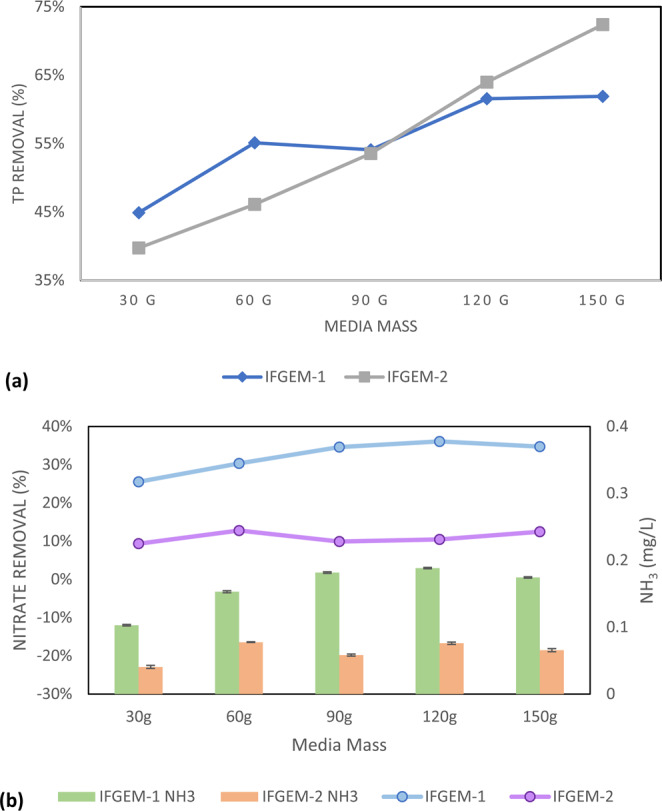
Outcome of the isotherm study: (**a**) the TP removals from the isotherm study of IFGEM-1 and IFGEM-2 under neutral pH condition and (**b**) the nitrate removal and ammonia generation from the isotherm study on IFGEM-1 and IFGEM-2 under neutral pH condition.

The isotherm study results for the phosphate adsorption of IFGEM-1 and IFGEM-2 under a neutral pH condition are shown in Fig. 3(a). IFGEM-1 tends to achieve higher TP removal at a lower mass ratio of media to liquid, while IFGEM-2 showed higher TP removal at a higher mass ratio of media to liquid. The Langmuir and Freundlich isotherm equation parameters of IFGEM-1 and IFGEM-2 are shown in Table 2S (Supplemental Materials 1). As most *1/q*_*m*_ values are negative in the Langmuir equation, it is inappropriate to apply for the calculation of the maximum absorption capacity (*q*_*m*_). Therefore, the Freundlich relative absorption capacity was selected from the Freundlich section in this study as the comparative basis.

The isotherm study results of IFGEM-1 and IFGEM-2 for nitrate reduction under a neutral pH condition, as well as the corresponding ammonia generation, are shown in Fig. 3(b). Ammonia generation was confirmed in both IFGEMs, but IFGEM-1 seemed to produce two to three times more ammonia when compared with IFGEM-2. Also, IFGEM-1 removed more nitrate (up to 35%) while IFGEM-2 only achieved approximately 10% nitrate removal. The Langmuir and Freundlich isotherm equation parameters of IFGEM-1 and IFGEM-2 are shown in Table 3S (Supplemental Materials 1). Since the *1/q*_*m*_ values were negative in the Langmuir equation, it suggestes it is not suitable for application in the calculation of the maximum absorption capacity (*q*_*m*_). Consequently, the Freundlich relative absorption capacity was selected.

### Column study

**Figure 4:**
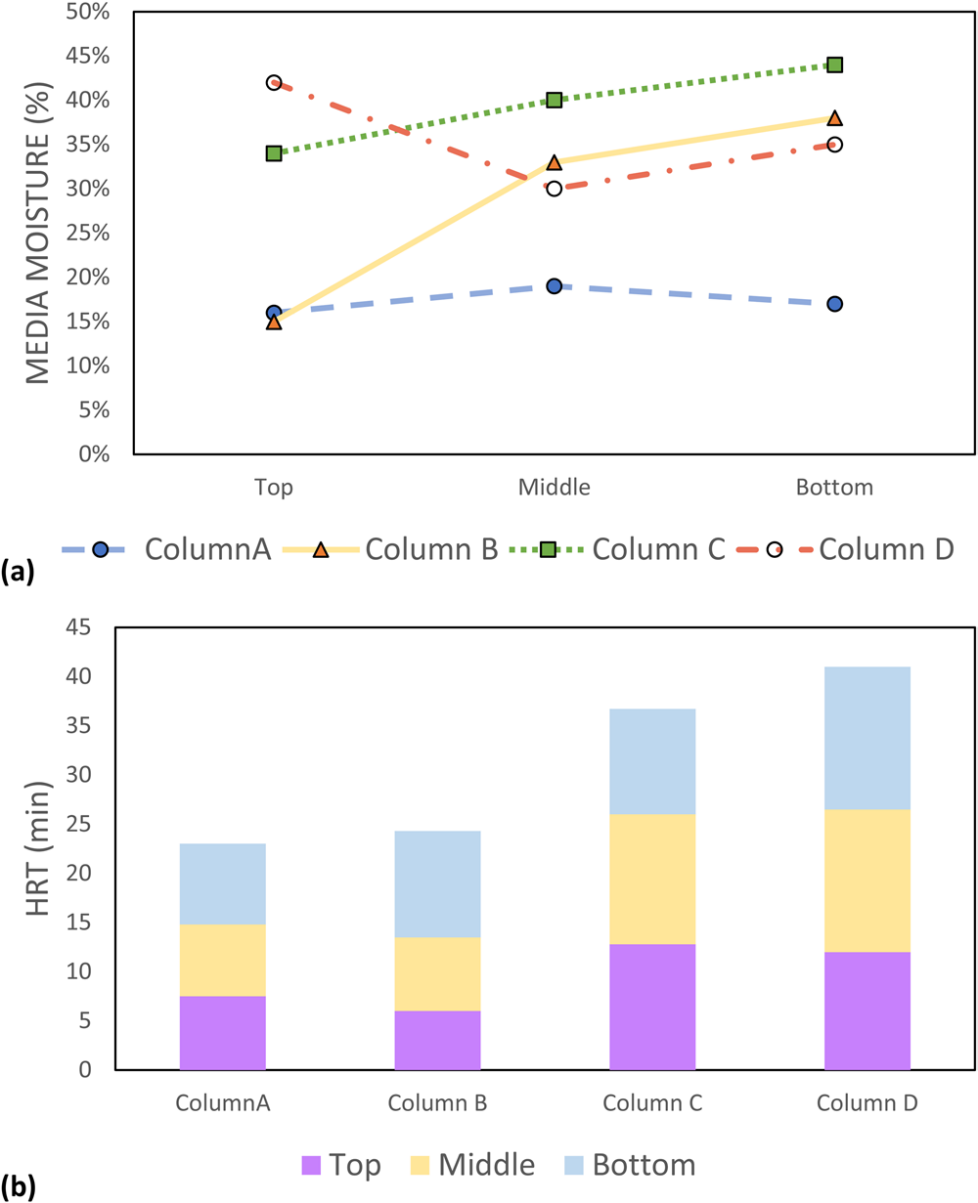
Outcome of the column study: (**a**) soil/media moisture content and (**b**) hydraulic retention time from each column section under operating condition.

The column study was designed to mimick real-world conditions. The measurements of pH, DO, and the ORP from the inlets and each sampling port of the columns are summarized in Table 4S (Supplemental Materials 1). In general, the ORP values of columns B and C decreased at the top section, then slightly increased and stabilized in the remaining two sections. However, the ORP trend in columns A and D decreased continuously from the top to the bottom section, which was particularly salient in column D. As the influent nutrient concentration increased, the ORP decreasing trend seemed to slow down in columns A and D. A significant DO decrease also occurred in columns A and D when compared to columns B and C, in which only slight DO increments were observed. For pH measurements, columns A and D exhibited trends of increasing pH values from the top to the bottom section, similar to the ORP measurements under all concentration levels. Column C showed steady pH values across all three sections. Further, the soil/media moisture and HRT of each section are summarized in Fig. 4. Column A and the first section of column B (IFGEM-1) showed lower moisture content, usually less than 20%. However, the remaining media exhibited much higher moisture contents, and the average moisture contents were 35.50%, 35.66%, and 39.3 3% for BAM, IFGEM-2, and natural soil, respectively.

#### Nitrate removal and ammonia generation

**Figure 5:**
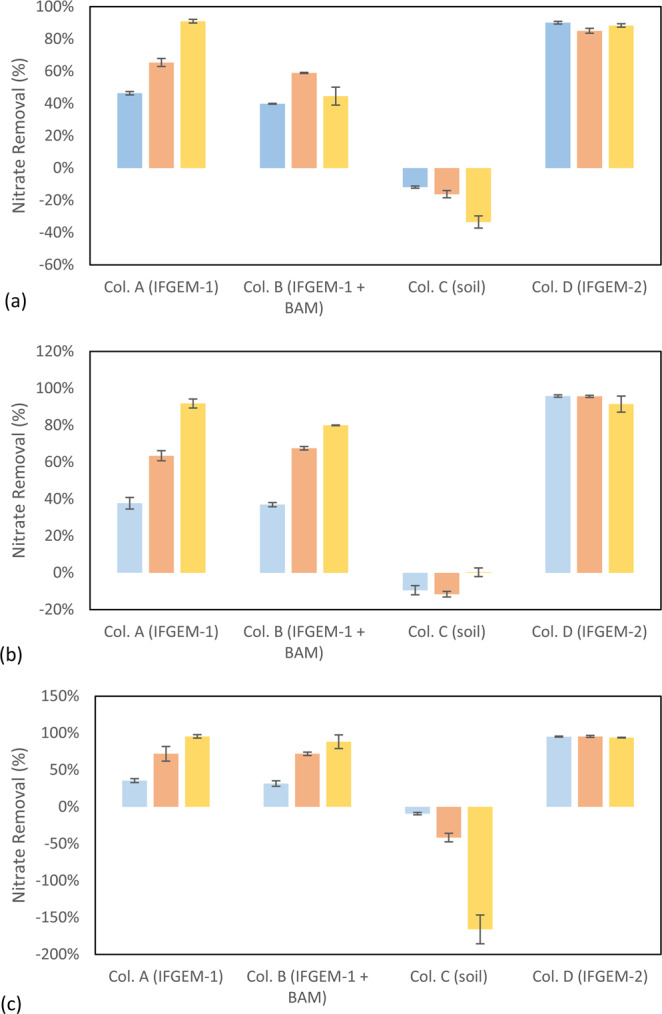
Outcome of the column study:cumulative nitrate removal at each section through columns when inlet nitrate concentration = (**a)** 0.6 mg/L, (**b**) 1.2 mg/L, and (**c**) 1.8 mg/L.

The cumulative nitrate removal at each sampling port of all columns is shown in Fig. 5 given the three different influent nitrate concentrations (denoted as levels 1 to 3). Nitrate removals were observed in columns A, B, and D, and only the control column C with natural soil showed negative or minor removal at all concentration levels. When the inlet nitrate concentration was 0.6 mg/L, columns A and D exhibited the highest nitrate removals of 91.01% and 88.32%, respectively. Column B showed a moderate nitrate removal of 44.56%. When the inlet nitrate concentration increased to 1.2 mg/L, the overall removals of column A and D were 91.76% and 91.43%, respectively, with column B achieving 79.95% nitrate removal. At the highest nitrate concentration of 1.8 mg/L, the overall removals of nitrate were up to 95.53% for column A, 94.49% for column D, and 75.85% for column B.

**Figure 6:**
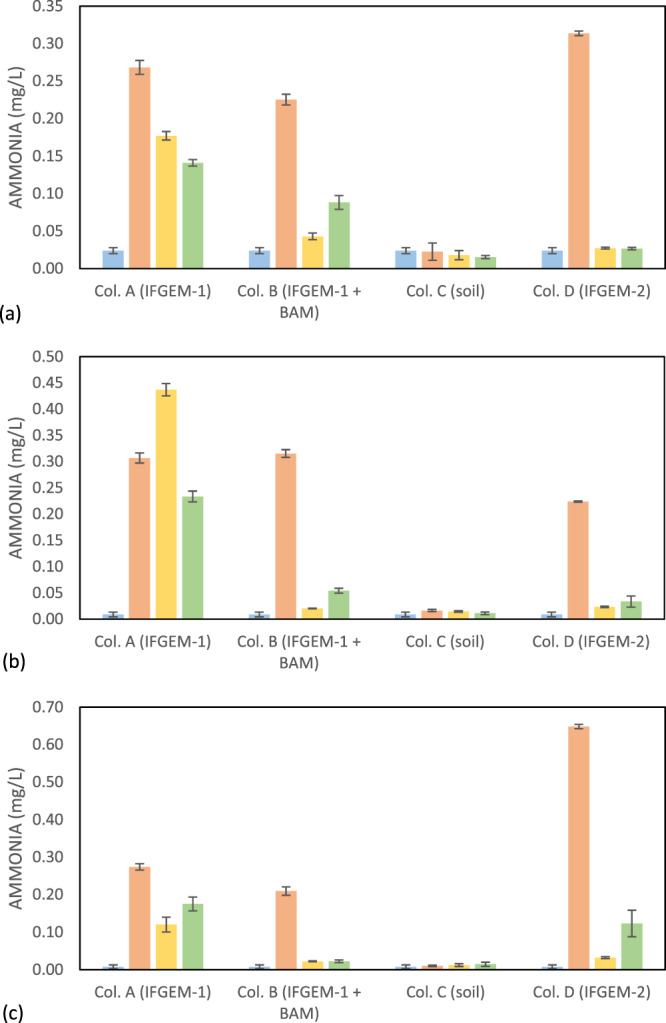
Outcome of the column study: ammonia concentrations at the outlet of each section through columns when inlet nitrate concentration = (**a**) 0.6 mg/L, (**b**) 1.2 mg/L, and (**c**) 1.8 mg/L.

The generation of ammonia from the IFGEM treatment process was confirmed as the byproduct of nitrate reduction. The ammonia concentrations from each sampling port under three different inlet nitrate concentration levels are shown in Fig. 6. Ammonia generation was positively related to the nitrate removal in the two IFGEM columns, particularly in the top sections, where the majority nitrate was removed. The higher the nitrate concentration in the influent, the more ammonia was produced. It is noticeable that the ammonia concentration of the treated effluent was 7 to 23 times higher than the influent values for column A. Column D exhibited some differences, as the first section generated a significant amount of ammonia, but the effluent from the bottom section showed negligible ammonia level. Thus, IFGEM-2 was able to remove most of the ammonia generated from the first section.

#### Phosphorus removal

**Figure 7:**
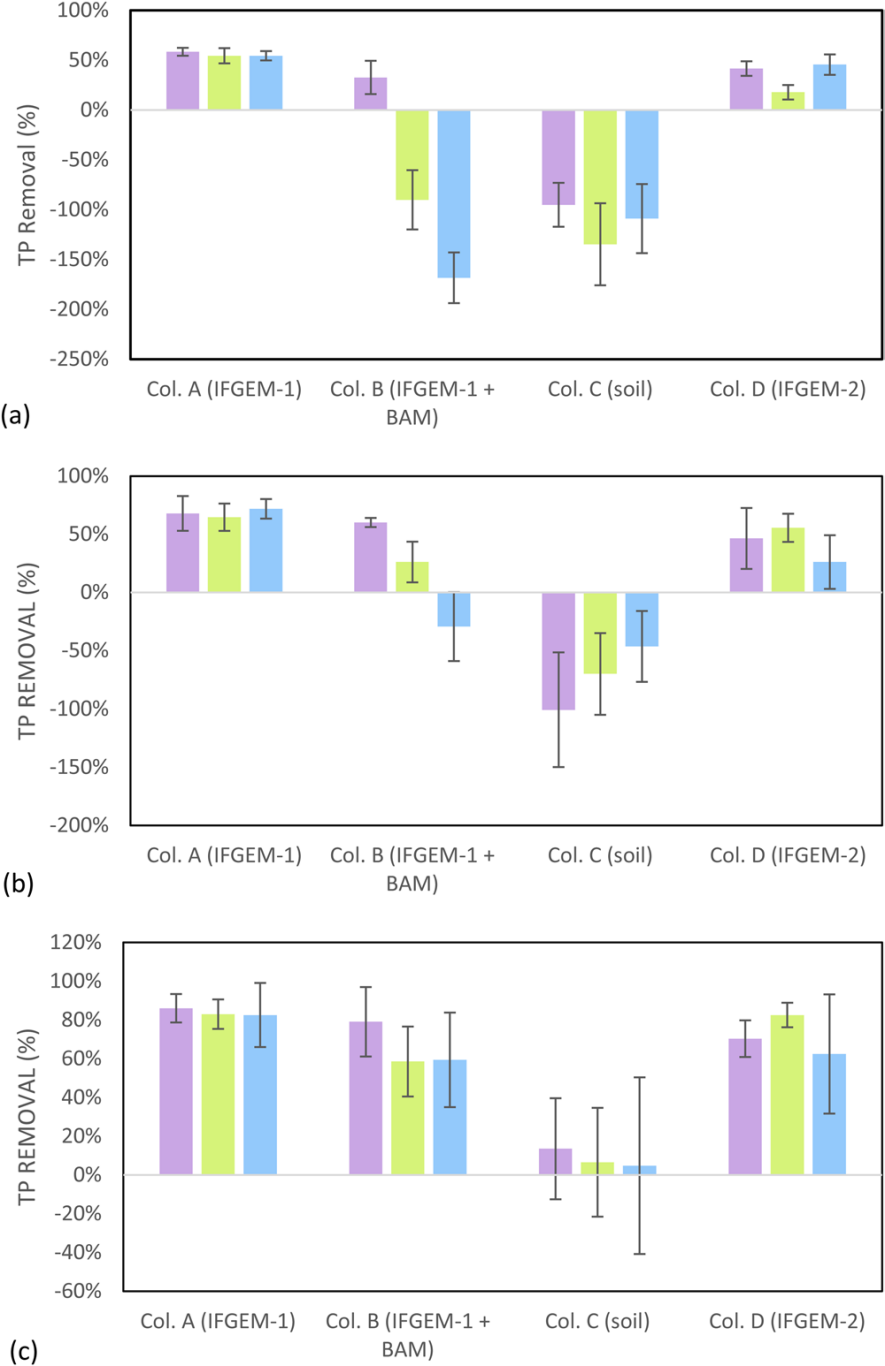
Outcome of the column study: total phosphorus removal when the inlet TP = (**a**) 0.3 mg/L, (**b**) 0.5 mg/L, and (**c**) 0.7 mg/L.

When the inlet TP = 0.3 mg/L, the overall TP removals of column A and D were 54.46% and 45.54%, respectively (Fig. 7). When the inlet TP concentration increased to 0.5 mg/L, the overall TP removal values were 71.90% and 26.14%, and the overall removals increased to 82.53% and 62.45% when the influent TP was 0.7 mg/L. In column B, the first section showed removal patterns similar to column A, but the following two sections exhibited negative removals under concentration levels 1 and 2 (-168.32% and -29.41%). A much higher TP removal of 59.39% was achieved under concentration level 3. However, the TP removals of column C (natural soil) were mostly negative or negligible under all concentration levels.

### Results of kinetics study

Nutrients cannot flow through the flasks during the isotherm test, but the adsorption of nutrients in the column test is a function of time, as the water flows through the column at different depths (Table 5S, Supplemental Materials 1). In column A, the kinetics analysis showed that IFGEM-1 was mainly represented by zero order, and the increase of influent nutrient concentration enhanced the reaction rate constant from 0.0258 to 0.0809 for nitrate reduction and from 0.0070 to 0.0242 for phosphorus removal. The situation was similar for column D (IFGEM-2), where zero order dominated the reaction kinetic and the rate constant also increased from 0.0027 to 0.0106 for phosphorus removal and from 0.0135 to 0.0388 for nitrate reduction as the influent concentration increased. In column B, the nitrate reduction mainly followed zero order, and the rate constant increased from 0.0124 to 0.0551. However, in column B both the nitrate reduction and phosphorus removal kinetics showed fluctuating reaction orders under various influent concentrations and sometimes with low R-squared values. The kinetics equation for column C cannot reflect any reaction order because all R-squared values under three influent conditions were low for both nitrate reduction and phosphorus removal.

### Results of ANOVA analysis

The two way ANOVA analysis^35^ was applied to test the null hypotheses for nitrate and TP removal separately. The p values from Table 6S (Supplemental Materials 1) can be viewed with 95% confidence for each paired column. Most of the p values fell within the rejection region, which means there were significant differences between each pair of columns in terms of nutrient removal. However, there were several exceptions: for nitrate removal there were no significant differences of the overall removal between columns A and D, nor were there significant differences between the interaction of influent concentrations and column types. Columns B and C also exhibited no significant differences in interaction regarding the column types and influent concentrations. For TP removal, only insignificant differences were found between the overall removals of columns B and C.

## Discussion

### Nitrate and ammonia removal mechanisms

**Figure 8:**
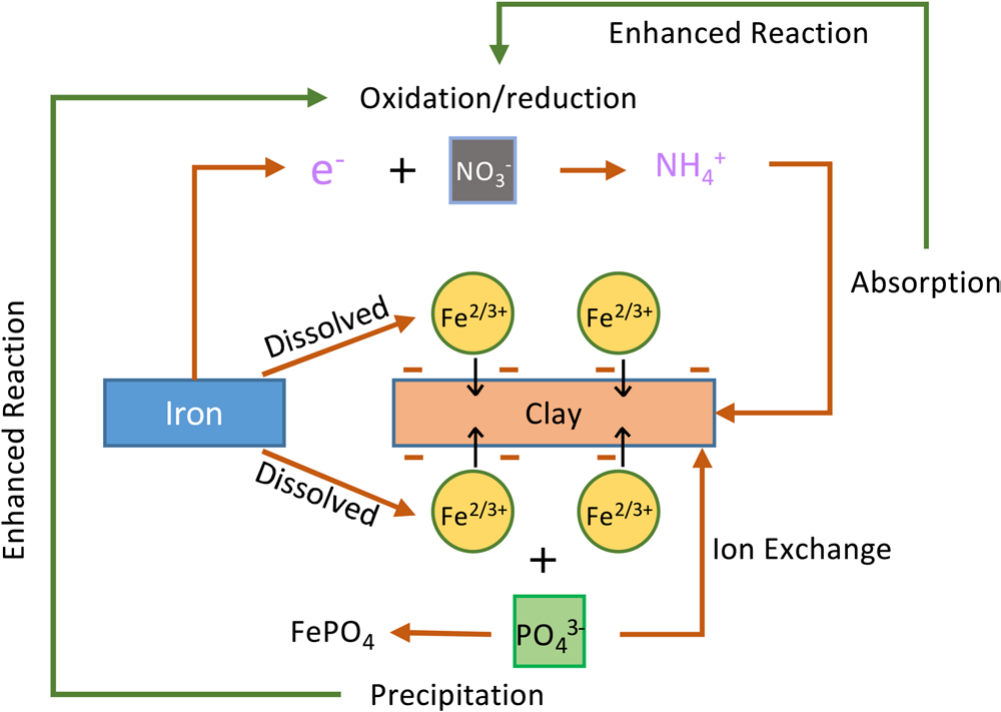
Schematic diagram of interactions between clay and soluble ions in the treatment process. Nitrate and phosphate are attracted by ferrous/ferric ions which are tied to the negatively charged clay surface. It forms the ion aggregation that accelerates the nitrate reduction and phosphorus precipitation with effective ammonia absorption.

The physicochemical interactions between nitrate and sorption media are directly related to the nitrate reduction process through IFGEMs, as both IFGEM columns (A and D) exhibited promising removal efficiencies at various influent nutrient concentrations when iron functioned as an electron donor. However, IFGEM-2 was more effective for nitrate removal when compared with IFGEM-1. IFGEM-2 was able to remove all the nitrate at the top section, but IFGEM-1 required using all three sections to achieve equivalent treatment (as indicated in Fig. 5). The main reason for this outcome is that IFGEM-2 contains clay, which aggregates the nitrate concentration through electromagnetic force (weak force)^36^ on its surface (anion attracted by the positively charged clay surface). Normally the surface of clay is negatively charged, but it can be reversed if the clay particles are modified with metal ions (ferrous and ferric ions in this study) that cover the surface of the clay and form a positively charged layer^37^, enhancing the clay’s anion exchange capacity. This further formed a mutual physical attraction between clay particles and iron filings that kept the clay close to the source of metal ions, enhancing the nitrate reduction process through direct contact with iron (Fig. 8). Dong *et al*.^38^ observed the same enhancement of nitrate removal with nano-size iron particles and clay. The main nitrate reduction reaction equation is shown in Eq. 4^39^. Other explanations might also be applicable here, such as that the clay content contributed to 4 times higher BET surface area in IFGEM-2 than IFGEM-1 (Table 2). IFGEM-2 achived almost doubled the HRT when compared with IFGEM-1, which potentially increased the ammonia removal, as well as nitrate reduction, with a longer contact time (Fig. 4). More intensive reactions in IFGEM-2 are evident from the significant decrease of ORP values in the column study when compared to IFGEM-1 (Tables 4S and 5S, Supplemental Materials 1). A similar phenomenon was observed by Ruangchainikom, *et al*.^39^ with zero valent iron involved nitrate reduction.

4 $$N{O}_{3}^{-}+4F{e}^{0}+10{H}_{3}{O}^{+}\to 4F{e}^{2+}+N{H}_{4}^{+}+13{H}_{2}O$$

It is indictive that the concentration of ammonia, the byproduct of nitrate reduction, was significantly lower in the effluent of IFGEM-2 than IFGEM-1 under all influent concentrations. This is likely due to the absence of clay content in IFGEM-1; clay is essential for providing cation exchange capacity for ammonia absorption^40^. In IFGEM-2, the intensive nitrate reduction (oxidation-reduction reaction) happened mainly at the top section (achieved over 90% nitrate removal, Fig. 5), which allowed the remaining two sections to perform mainly ammonia absorption/adsoprtion, whereas IFGEM-1 was not capable of achieving such removal in only one section. This is evident from the ammonia concentration and nitrate removal over each sampling port in Figs. 5 and 6. Additionally, the ammonia produced by IFGEM-1 at the top section of column B was also successfully removed by BAM in subsequent sections, which evidenced the clay’s contribution to ammonia removal. On the other hand, the removal of ammonia impacts the nitrate reduction reaction (Eq. 4) equilibrium as well, since the decrease of product’s concentration will shift the reaction equilibrium to reduce more nitrate.

The isotherm study result appeared inconsistent with the column study, since IFGEM-1 showed better removal than IFGEM-2 and produced more ammonia; however, this inconsistency was caused by differing hydraulic conditions. Nitrate reduction requires close contact between iron and nitrate, which was not the case in the isotherm study, in which the nitrate ions were attracted by clay particles, but were not given much opportunity for contact with the iron, as the solution was shaken on a rotary platform. All particles were moving around, whereas they were much more stable in their positions in the column study.

### Comparison of phosphorus and nitrate removal mechanisms

When simultaneous removal of nitrate and phosphorus can be accomplished, the synergetic effects among media can be realized fully, as shown in Fig. 8. IFGEM-1 showed higher and more stable phosphorus removal than IFGEM-2, because the clay minerals in IFGEM-2 can aggregate ferrous/ferric ion onto their surface areas, whereas the ferrous/ferric ions had more freedom in the liquid phase in IFGEM-1 for precipitating the phosphate ion into $FeP{O}_{4}$ or $F{e}_{3}{(P{O}_{4})}_{2}$ (Eqs. 5 and 6)^41^. As the influent nitrate and phosphorus concentration increased, more ferrous/ferric ion was generated in the IFGEMs through nitrate reduction. As a result, the reaction equilibrium of Eqs. 5 and 6 can be shifted to precipitate more phosphorus (Fig. 7). The facilitated phosphorus precipitation can also be observed from reaction kinetics, where phosphorus removal rate constants showed similar or higher improvement than the nitrate removal rate constants when the nitrate concentrations increased by 2 to 3 times, while the TP concentration increased only up to 2.3 times at maximum.

Also, based on the isotherm study result (Fig. 3), IFGEM-2 showed higher TP removals when more media mass was available but less TP removal when it was not, compared to IFGEM-1. Ferrous/ferric ions tend to be aggregated on the surface of the clay, and more media increases the likelihood of the phosphate making contact with ferrous/ferric ions for precipitation at a faster pace, triggering the aggregated ferrous/ferric concentration on the clay surface. However, the free ferrous/ferric ions were dissolved in a liquid phase in IFGEM-1 for precipitating phosphorus. This is why TP removal increased more significantly in IFGEM-2 than in IFGEM-1 when increasing the same amount of media mass. Additionally, IFGEM-1 performed better in TP removal within the column study according to the ORP values, because IFGEM-1 had a better balance between the ferrous/ferric ions generation and ORP consumption for phosphorus removal (Table 4S, Supplemental Materials 1)^42^.

5 $$F{e}^{3+}+P{O}_{4}^{3-}\to FeP{O}_{4}\downarrow $$

6 $$3F{e}^{2+}+2P{O}_{4}^{3-}\to F{e}_{3}{(P{O}_{4})}_{2}\downarrow $$

### Nutrient recovery potential

From the mophological analysis result shown in Fig. 2, the iron filings in IFGEMs were dissolved and coated by surrounding particles during the treatment. Presumably, once the iron filings have completely dissolved into surrounding particles, the media should be considered exhausted and due for replacement. However, the used or spent IFGEM can be considered as alternative nutrients resource for soil amendment, since the nutrients have been well retained in the media via the treatment process. Therefore, the spent IFGEMs do have some potential for application for nutrient recovery from nonpoint source pollution in urban stormwater runoff, agricultural discharge, and wastewater effluent.

## Conclusion

It has historically been difficult to control nonpoint source contamination originating from stormwater runoff and agricultural effluent. As this contamination negatively affects both sources of drinking water and agricultural production, its unfettered continuation amounts to a significant threat to the food security of the world. Hence, two IFGEM recipes were invented and evaluated in parallel for their nutrient removal performance in comparison against traditional green sorption media (BAM) and natural soil collected from a study site in Ocala, Florida. Both IFGEM recipes showed excellent nitrate reduction due to the existence of iron filings as the reactive electron donor, but the mix of IFGEM-1 was less capable of removing ammonia (byproduct), while the mix of IFGEM-2 was able to remove most generated ammonia. The phosphorus removal was enhanced by the nitrate reduction in two IFGEM-only columns, given that the iron oxides produced from iron filings can bond with phosphorus for precipitation; however, IFGEM-1 showed superior and more stable phosphorus removal than IFGEM-2 because the ferrous/ferric ions were dissolved in the solution rather than aggregated on the clay surface, and the ORP decrement in IFGEM-1 was not as influential as that in IFGEM-2.

Overall, it is indictive that spent IFGEMs could be candidate media mixes for nutrient recovery/reuse when ammonia and phosphorus are kept within IFGEM mixes under various changing influent conditions. The mix of IFGEM-1 is preferred for treatments for higher phosphorus removal, albeit lower ammonia removal and recovery. The mix of IFGEM-2 is appropriate for treating stormwater runoff, agricultural discharge, and wastewater effluents with the simultaneous removal of phosphorus and nitrate, albeit with lower phosphorus removal. Nevertheless, both IFGEM recipes showed possible nutrient recovery potential as soil amendment through different landscape environments. Future work may be directed to assess the treatability of IFGEMs under various temperature and pH conditions to ensure that IFGEMs can be applied in different scenarios with quantitative information regarding the amount of nutrients that can be recovered from spent IFGEMs.

## Supplementary information

Supplementary information.

## Data Availability

The raw data obtained from this study are available via a shared link (https://drive.google.com/file/d/15lV9HR3MkBEdwEtd30_WcT0KxOyo4Ow9/view?usp=sharing).
