# Supplementary material for: Assessing Nutrient Removal in Stormwater Runoff for Urban Farming with Iron filings-based Green Environmental Media
Source: Sci Rep. 2020 Jun 10;10:9379. doi: 10.1038/s41598-020-66159-7 (PMC7287050; doi:10.1038/s41598-020-66159-7)
Supplement: Supplementary file 1 — Supplementary information. [file 41598_2020_66159_MOESM1_ESM.docx]

**Assessing Nutrient Removal in Stormwater Runoff for Urban Farming with Iron filings-based Green Environmental Media**

Dan Wen, Ni-Bin Chang^*^, and Martin P. Wanielista

Department of Civil, Environmental, and Construction Engineering,

University of Central Florida, Orlando, FL 32816, USA

## Material Characterization

Two iron filings-based green environmental media (IFGEM) recipes and one biosorption activated media (BAM) recipe were selected in this study; media mix 1 is named IFGEM-1, and it consists of 96.2% fine sand 3.8% grinded iron filings (by volume). Media mix 2 is named IFGEM-2, and it consists of 80% sand, 10% tire crumb, 5% pure clay, and 5% grinded iron filings (by volume). Media mix 3 is BAM, and it is composed of 85% poorly graded sand, 10% tire crumb, and 5% clay (by volume), in which the tire crumb used are recyclable with no metal contents and mined clay has no less than 99% clay content. As the control media (media #4) in the experiment, natural soil was collected from SR 35 Basin 2 in Silver Springs watershed, located in Ocala, Florida.

ASTM Standard Practices are international standards that have been widely accepted and used for many materials, products, and systems. They were used to determine the particle size distribution, specific gravity, and Brunauer–Emmett–Teller surface area. In this study, ASTMD 422 was adopted for particle size distribution, ASTMD 854 was applied for specific gravity and micrometrics, and ASAP 2020 was applied for BET surface area. Media mixes 1 to 4 were tested with the methods mentioned above by a certified laboratory (EMSL, Inc.). Olympus LEXT OLS 3000 Confocal Scanning Microscope was used to characterize the microstructure changes of media mixes before and after the nutrient adsorption. This was conducted at the Advanced Materials Processing and Analysis Center at the University of Central Florida. The plane resolution could be as high as 0.12 µm, with simultaneous 3D and “true color” image acquisition. The media porosity was tested by pouring a known volume of water to a certain volume of media until the media mix was fully saturated. The infiltration rate was tested through the constant head method; and the media depth, density, cross-section area, and the time consumed for a certain volume of water to flow through the media were documented.

## Statistical Analysis

In order to determine if there are significant differences between overall nutrient removal efficiencies across different columns under various influent conditions, a two-way ANOVA analysis was performed with the aid of Microsoft Excel packages. This analysis of variance may determine if manipulating the influent concentration and switching to different green sorption media can create significant differences of nutrient removal. Each ANOVA analysis was considered statistically significant at a confidence interval of 95% (α = 0.05).

The comparison was made possible in pairs of two columns each time. There are two independent variables, including column number and influent concentration, given that different columns have different media for nutrient removal under varying influent concentrations. Based on triplicate samples, the p-value results associated with the ANOVA analysis may indicate if there is a significant difference in nutrient removal when the columns and the inlet conditions vary, as well as if there is significant interaction between the two variables. The first null hypothesis is H_0_: the means of nutrients removal grouped by the columns are the same; the second null hypothesis is H_0_: the means of nutrients removal grouped by the inlet conditions are the same; and the third null hypothesis is H_0_: there is no interaction between columns and inlet conditions.

## Appendix:


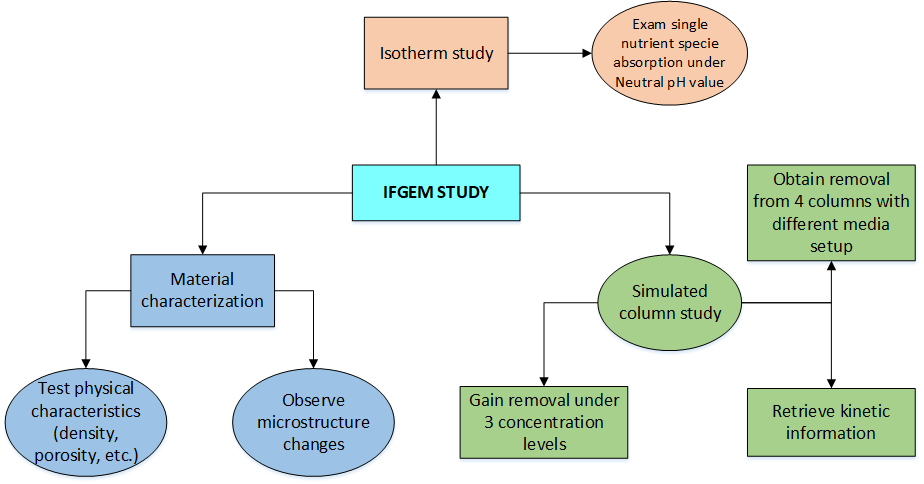


**Figure 1S. The flowchart for the current part of IFGEM study**

**Table 1S. Summary of previous studies of nutrient sorption media**

| Media used | Treated water | Nutrient removal | Limitation | Reference |
| --- | --- | --- | --- | --- |
| Phosphate precipitation with FeCl_3_·6H_2_O | Municipal wastewater with 0 – 5 mg/L OP_4_-P | 63% phosphate removal at 1:1 molar addition of Fe(III) at pH = 4 | Excessive addition of FeCl_3_·6H_2_O is necessary for ideal removal;  High maintenance cost | Fytianos, Voudrias et al. (1998) |
| Iron-aluminum hydroxyl (oxide) coated filter media | Artificial groundwater, tap water, treated wastewater with 0.5 mg/L OP_4_-P | Over 90% phosphate removal | Lack of ability for nitrogen species removal;  Special pre-treatment of the media is required | Ayoub, Koopman et al. (2001) |
| Bioretention system with soil, mulch, and plants | Urban stormwater runoff with 2-4 mg/L TN and 0.6 mg/L TP | > 90% heavy metal;  60 – 80% phosphorus;  Moderate TKN | Little nitrate was removed; Nitrate production confirmed | Davis, Shokouhian et al. (2001) |
| Iron packed bed in columns | Groundwater spiked with nitrate solution to 5 mg/L | > 80% nitrate removal | By-products generation of ammonia and metal ions | Westerhoff and James (2003) |
| Soil, sand, mulch in bioretention system with layer configurations | Synthetic water with 2 – 3 mg/L of nitrate, ammonia and phosphorus | 68% ammonia removal;  85% phosphorus removal; | Nitrate export was confirmed | Hsieh, Davis et al. (2007);  Hsieh, Davis et al. (2007) |
| Sand, tire crumb, sawdust, and limestone in different configurations | Stormwater with 0.38 - 2.5 mg/L nitrate, 0.125 – 0.785 mg/L OP_4_-P | Over 90% nitrate removal;  50 – 90% orthophosphate removal | Ammonia export was confirmed | Moberg (2008) |
| Expanded clay, tire crumb, sawdust, and lime stone in wetland system | Septic tank effluent with 40 – 80 µg/L TN | 75.4% TN removal;  94.9% TP removal | Lack of pathogen removal;  Limited nitrification process | Xuan, Chang et al. (2009) |
| Sand, limestone, sawdust, and tire crumb for stormwater dry ponds | Stormwater with 35 – 54 mg/L TN, 7.5 mg/L TP | 65 – 95% nitrate removal;  Completely ammonia removal | Certain requirement of media thickness for achieving proper HRT | Hossain, Chang et al. (2010) |
| Fine sand, tire crumb, and sawdust placed in vadose zone | Septic tank effluent with 1.76 mg/L ammonia, 0.352 nitrate, 0.116 nitrite, 1.498 OP_4_-P | 55% nitrate removal;  89% TP removal | Larger aerobic zone and longer HRT required for complete denitrification | Xuan, Chang et al. (2010) |
| Nanoscale zero valent iron supported on pillared clay | Synthetic solution with 0.806 mmol/L nitrate | Over 90% nitrate removal within 40 min of HRT | End products of ammonium was observed | Zhang, Li et al. (2011) |
| Fine sand, tire crumb, sawdust, and limestone in various configurations | Stormwater under various temperatures | 70 – 90% nitrate removal;  40 – 85% orthophosphate | Lower temperature may inhibit the nutrient removal | Chang, Wanielista et al. (2011) |
| Nanoscale zero valent iron | Synthetic phosphorus solution with 1 – 10 mg/L OP_4_-P | 96 – 100% removal with ~ 78% phosphorus recovery | Too expensive to conduct economic implication | Almeelbi and Bezbaruah (2012) |
| Tire crumb, silt, clay, and sand in stormwater infiltration basin | Stormwater with 0.46 – 0.90 mg/L TDP | Minor nitrate removal;  70% phosphorus removal | Enhanced denitrification is required for better removal effects | O'Reilly, Wanielista et al. (2012) |
| Cement sand, tire crumb, fine expanded clay, and limestone | Stormwater with 0.5 – 1.0 mg/L TDP | Maximum adsorption equilibrium is 0.0151 mg-P/g-absorbent | Lack of nutrients recovery potential | Jones, Chang et al. (2015) |

**Table 2S. Phosphorus Absorption Parameters of the Langmuir and Freundlich isotherm for IFGEM 1 and 2 under neutral pH condition**

| **IFGEM recipes** | **Isotherm equation for Langmuir** | **R-square value** | **1/(q_m_K_ads_)** | **1/q_m_ (mg/g)** | |
| --- | --- | --- | --- | --- | --- |
| IFGEM-1 | y = 628.74x - 938.59 | 0.8352 | 628.74 | -938.59 | |
| IFGEM-2 | y = 190.75x + 52.554 | 0.7635 | 190.75 | 52.554 | |
| **IFGEM recipes** | **Isotherm equation for Freundlich** | **R-square value** | **1/n** | **Log K** | **K (mg^1-(1/n)^ L^1/n^ g^-1^)** |
| IFGEM-1 | y’ = 3.1346x’ - 1.5403 | 0.86 | 3.1346 | -1.5403 | 0.0288 |
| IFGEM-2 | y’ = 1.0972x’ - 2.2837 | 0.7342 | 1.0972 | -2.2837 | 0.0052 |
| $x = 1/C_{e}; y = 1/q_{e}$. Where $C_{e}$ is the aqueous concentration of phosphorus (mg/L) and$q_{e}$ is the phosphorus concentration sorbed on the media (mg/g).  $x’ = log(C_{e}); y’ = log(q_{e})$. Where $C_{e}$ is the aqueous concentration of phosphorus (mg/L) and $q_{e}$ is the phosphorus concentration sorbed on the media (mg/g). | | | | | |

**Table 3S. Nitrate absorption parameters of the Langmuir and Freundlich isotherm for IFGEM 1 and 2 under neutral pH condition**

| **IFGEM recipes** | **Isotherm equation for Langmuir** | **R-square value** | **1/(q_m_K_ads_)** | **1/q_m_ (mg/g)** | |
| --- | --- | --- | --- | --- | --- |
| IFGEM-1 | y = 3754.5x - 4216.4 | 0.7158 | 3754.5 | -4216.4 | |
| IFGEM-2 | y = 4924.3x - 4456.2 | 0.1362 | 4924.3 | -4456.2 | |
| **IFGEM recipes** | **Isotherm equation for Freundlich** | **R-square value** | **1/n** | **Log K** | **K (mg^1-(1/n)^ L^1/n^ g^-1^)** |
| IFGEM-1 | y’ = 7.3878x’ - 1.9373 | 0.8565 | 7.3878 | -1.9373 | 0.0116 |
| IFGEM-2 | y’ = 7.7666x’ - 3.2904 | 0.0538 | 7.7666 | -3.2904 | 0.0005 |
| $x = 1/C_{e}; y = 1/q_{e}$. Where $C_{e}$ is the aqueous concentration of nitrate (mg/L) and $q_{e}$ is the nitrate concentration sorbed on the media (mg/g).  $x’ = log(C_{e}); y’ = log(q_{e})$. Where $C_{e}$ is the aqueous concentration of nitrate (mg/L) and $q_{e}$ is the nitrate concentration sorbed on the media (mg/g). | | | | | |

**Table 4S. Average ORP, dissolved oxygen, and pH values in the column study**

| **Column** | **Port** | **Inlet = 0.6 mg/L nitrate** | | | **Inlet = 1.2 mg/L nitrate** | | | **Inlet = 1.8 mg/L nitrate** | | |
| --- | --- | --- | --- | --- | --- | --- | --- | --- | --- | --- |
|  |  | ORP | DO | pH | ORP | DO | pH | ORP | DO | pH |
|  | inlet | 327.50 | 8.76 | 6.98 | 316.40 | 7.58 | 7.10 | 320.17 | 8.35 | 6.76 |
|  | Port 1 | 154.57 | 7.99 | 8.34 | 232.77 | 8.24 | 8.14 | 180.53 | 6.86 | 8.52 |
| A | Port 2 | 214.43 | 7.41 | 8.26 | 237.93 | 7.64 | 8.05 | 244.20 | 8.43 | 7.88 |
|  | Outlet | 122.37 | 6.09 | 8.85 | 184.30 | 6.52 | 8.89 | 201.43 | 6.64 | 8.82 |
|  | Port 1 | 117.53 | 8.21 | 9.67 | 165.70 | 8.51 | 9.37 | 99.53 | 7.01 | 9.44 |
| B | Port 2 | 243.33 | 8.57 | 8.00 | 256.87 | 8.59 | 7.87 | 240.23 | 8.52 | 7.85 |
|  | Outlet | 235.07 | 8.26 | 7.96 | 265.03 | 8.64 | 7.99 | 296.27 | 8.07 | 7.81 |
|  | Port 1 | 245.37 | 8.85 | 8.33 | 213.60 | 8.63 | 8.42 | 221.93 | 8.76 | 8.44 |
| C | Port 2 | 240.20 | 8.85 | 8.26 | 219.83 | 8.60 | 8.38 | 247.63 | 8.84 | 8.28 |
|  | Outlet | 246.17 | 8.74 | 8.23 | 253.93 | 8.92 | 8.33 | 257.60 | 8.86 | 8.30 |
|  | Port 1 | 246.83 | 8.54 | 8.08 | 268.80 | 7.79 | 7.67 | 271.23 | 8.32 | 7.59 |
| D | Port 2 | -61.40 | 7.10 | 8.69 | 97.23 | 7.76 | 8.07 | 57.23 | 8.01 | 8.20 |
|  | Outlet | -102.90 | 4.98 | 7.77 | 56.97 | 5.28 | 8.47 | 0.03 | 7.01 | 8.39 |

**Table 5S. Kinetics information of each column under various influent conditions**

| column | Nutrient species | Concentration level 1 | R^2^ / reaction order | Concentration level 2 | R^2^ / reaction order | Concentration level 3 | R^2^ / reaction order |
| --- | --- | --- | --- | --- | --- | --- | --- |
| A | Nitrate | y = -0.0258x + 0.6898 | 0.9593 / Zero | y = -0.0486x + 1.1884 | 0.9907 / Zero | y = -0.0809x + 1.8616 | 0.9744 / Zero |
|  | Phosphorus | y = -0.007x + 0.2746 | 0.5301 / Zero | y = -0.0141x + 0.4086 | 0.632/ Zero | y = -0.0242x + 0.5572 | 0.5538 / Zero |
| B | Nitrate | y = -0.0124x + 0.6138 | 0.4775 / Zero | y = 0.1372x + 0.6571 | 0.9896 / 2^nd^ | y = -0.0551x + 1.8511 | 0.9602 / Zero |
|  | Phosphorus | y = 0.027x + 0.2311 | 0.8537 / Zero | y = 0.0097x + 0.3317 | 0.2713 / Zero | y = -0.0128x + 0.5274 | 0.2632 / Zero |
| C | Nitrate | y = -0.0085x + 1.3361 | 0.9541 / 2^nd^ | y = 0.0003x + 1.2911 | 0.0049 / Zero | y = -0.0041x + 0.5331 | 0.8004 / 2^nd^ |
|  | Phosphorus | y = 0.0103x + 0.4281 | 0.6830 / Zero | y = -0.0142x + 1.6263 | 0.2803 / 2^nd^ | y = -0.0005x + 0.7253 | 0.0347 / Zero |
| D | Nitrate | y = -0.0135x + 0.5232 | 0.5389 / Zero | y = -0.0236x + 0.8296 | 0.518 / Zero | y = -0.0388x + 1.3747 | 0.6689 / Zero |
|  | Phosphorus | y = -0.0027x + 0.3015 | 0.4374 / Zero | y = -0.003x + 0.406 | 0.1786 / Zero | y = -0.0106x + 0.5627 | 0.4470 / Zero |
| Zero, 1^st^, and 2^nd^ represent zero, first, and second order reactions, x = reaction time, equivalent to HRT in column study; y = nutrient concentration (C) in effluent for Zero order reaction, ln(C) for 1^st^ order reaction, and 1/C for 2^nd^ order reaction. | | | | | | | |

**Table 6S. ANOVA analysis between paired columns with three inlet conditions**

| **compared aspects** | **A-B** | **A-C** | **A-D** | **B-C** | **B-D** | **C-D** |
| --- | --- | --- | --- | --- | --- | --- |
|  | Nitrate removal analysis | | | | | |
| **Columns** | $2.76\times{10}^{-9}$ | $2.87\times{10}^{-17}$ | 0.1208 | $9.30\times{10}^{-14}$ | $7.22\times{10}^{-9}$ | $5.64\times{10}^{-17}$ |
| **Inlet conditions** | $1.37\times{10}^{-6}$ | $1.91\times{10}^{-7}$ | 0.0005 | $1.55\times{10}^{-8}$ | $1.07\times{10}^{-6}$ | $1.74\times{10}^{-7}$ |
| **Interaction** | $5.67\times{10}^{-6}$ | $1.05\times{10}^{-6}$ | 0.7538 | 0.7332 | $1.58\times{10}^{-5}$ | $3.44\times{10}^{-6}$ |
|  | Phosphorus removal analysis | | | | | |
| **Columns** | $4.67\times{10}^{-10}$ | $7.62\times{10}^{-9}$ | $2.35\times{10}^{-5}$ | 0.6725 | $1.92\times{10}^{-8}$ | $1.75\times{10}^{-7}$ |
| **Inlet conditions** | $5.57\times{10}^{-9}$ | $5.66\times{10}^{-5}$ | $3.02\times{10}^{-4}$ | $4.47\times{10}^{-8}$ | $2.55\times{10}^{-8}$ | 0.0002 |
| **Interaction** | $9.49\times{10}^{-8}$ | 0.0039 | $5.35\times{10}^{-3}$ | 0.0026 | $9.25\times{10}^{-8}$ | 0.0014 |

## Reference

Almeelbi, T. and A. Bezbaruah (2012). "Aqueous phosphate removal using nanoscale zero-valent iron." Journal of Nanoparticle Research **14**(7): 900.

Ayoub, G. M., B. Koopman and N. Pandya (2001). "Iron and aluminum hydroxy (oxide) coated filter media for low-concentration phosphorus removal." Water Environment Research **73**(4): 478-485.

Chang, N. B., M. P. Wanielista and D. Henderson (2011). "Temperature effects on functionalized filter media for nutrient removal in stormwater treatment." Environmental Progress & Sustainable Energy **30**(3): 309-317.

Davis, A. P., M. Shokouhian, H. Sharma and C. Minami (2001). "Laboratory study of biological retention for urban stormwater management." Water Environment Research: 5-14.

Fytianos, K., E. Voudrias and N. Raikos (1998). "Modelling of phosphorus removal from aqueous and wastewater samples using ferric iron." Environmental Pollution **101**(1): 123-130.

Hossain, F., N. B. Chang and M. Wanielista (2010). "Modeling kinetics and isotherms of functionalized filter media for nutrient removal from stormwater dry ponds." Environmental Progress & Sustainable Energy **29**(3): 319-333.

Hsieh, C.-h., A. P. Davis and B. A. Needelman (2007). "Bioretention column studies of phosphorus removal from urban stormwater runoff." Water Environment Research **79**(2): 177-184.

Hsieh, C.-h., A. P. Davis and B. A. Needelman (2007). "Nitrogen removal from urban stormwater runoff through layered bioretention columns." Water Environment Research **79**(12): 2404-2411.

Jones, J., N.-B. Chang and M. P. Wanielista (2015). "Reliability analysis of nutrient removal from stormwater runoff with green sorption media under varying influent conditions." Science of the Total Environment **502**: 434-447.

Moberg, M. (2008). "The Effectiveness of Specifically Designed Filter Media to Reduce Nitrate and Orthophosphate in Stormwater Runoff."

O'Reilly, A. M., M. P. Wanielista, N.-B. Chang, Z. Xuan and W. G. Harris (2012). "Nutrient removal using biosorption activated media: Preliminary biogeochemical assessment of an innovative stormwater infiltration basin." Science of the Total Environment **432**: 227-242.

Westerhoff, P. and J. James (2003). "Nitrate removal in zero-valent iron packed columns." Water Research **37**(8): 1818-1830.

Xuan, Z., N.-B. Chang, A. Daranpob and M. Wanielista (2009). "Initial test of a subsurface constructed wetland with green sorption media for nutrient removal in on-site wastewater treatment systems." Water Quality, Exposure and Health **1**(3-4): 159-169.

Xuan, Z., N.-B. Chang, M. Wanielista and F. Hossain (2010). "Laboratory-scale Characterization of a green sorption medium for on-site sewage treatment and disposal to improve nutrient removal." Environmental Engineering Science **27**(4): 301-312.

Zhang, Y., Y. Li, J. Li, L. Hu and X. Zheng (2011). "Enhanced removal of nitrate by a novel composite: nanoscale zero valent iron supported on pillared clay." Chemical Engineering Journal **171**(2): 526-531.
